# Heritability and polygenic load for comorbid anxiety and depression

**DOI:** 10.1038/s41398-025-03325-3

**Published:** 2025-03-27

**Authors:** Fara Tabrizi, Jörgen Rosén, Hampus Grönvall, Victor Rahimzadeh William-Olsson, Erik Arner, Patrik KE Magnusson, Camilla Palm, Henrik Larsson, Alexander Viktorin, Jens Bernhardsson, Johanna Björkdahl, Billy Jansson, Örjan Sundin, Xuan Zhou, Doug Speed, Fredrik Åhs

**Affiliations:** 1 Department of Psychology and Social Work, Mid Sweden University, Ostersund, Sweden; 2 Department of Clinical Neuroscience, Karolinska Institute, Stockholm, Sweden; 3 Department of Medicine, Karolinska Institute, Stockholm, Sweden; 4 Department of Medical Epidemiology and Biostatistics, Karolinska Institute, Stockholm, Sweden; 5 Swedish Twin Registry, Karolinska Institute, Stockholm, Sweden; 6 School of Medical Sciences, Örebro University, Örebro, Sweden; 7 Centre for Quantitative Genetics and Genomics, Aarhus University, Aarhus, Denmark

**Keywords:** Depression, Predictive markers, Clinical genetics, Personalized medicine

## Abstract

Anxiety and depression commonly occur together resulting in worse health outcomes than when they occur in isolation. We aimed to determine whether the genetic liability for comorbid anxiety and depression was greater than when anxiety or depression occurred alone. Data from 12,792 genotyped twins (ages 38–85) were analysed, including 1,986 complete monozygotic and 1,594 complete dizygotic pairs. Outcomes were prescription of antidepressant and anxiolytic drugs, as defined by the World Health Organization Anatomical Therapeutic Chemical Classification System (ATC) convention, for comorbid anxiety and depression (*n* = 1028), anxiety only (*n* = 718), and depression only (*n* = 484). Heritability of each outcome was estimated using twin modelling, and the influence of common genetic variation was assessed from polygenic scores (PGS) for depressive symptoms, anxiety, and 40 other traits. Heritability of comorbid anxiety and depression was 79% compared with 41% for anxiety and 50% for depression alone. The PGS for depressive symptoms likewise predicted more variation in comorbid anxiety and depression (adjusted odds ratio per *SD* PGS = 1.53, 95% CI = 1.43–1.63; Δ*R*^2^ = 0.031, ΔAUC = 0.044) than the other outcomes, with nearly identical results when comorbid anxiety and depression was defined by International Classification of Diseases (ICD) diagnoses (adjusted odds ratio per *SD* PGS = 1.70, 95% CI = 1.53–1.90; Δ*R*^2^ = 0.036, ΔAUC = 0.051). Individuals in the highest decile of PGS for depressive symptoms had over 5 times higher odds of being prescribed medication for comorbid anxiety and depression compared to those in the lowest decile. While results on a predominant role of depressive symptoms may have been biased by the size and heterogeneity of available data bases, they are consistent with the conclusion that genetic factors explain substantially more variation in comorbid anxiety and depression than anxiety or depression alone.

## Introduction

Anxiety- and mood disorders are the most commonly occurring psychiatric disorders in the general population, with lifetime prevalence estimates of up to 33.7% and 14.7%, respectively [1, 2]. Symptoms of these disorder categories most often occur together, as evident by nearly half of adults with anxiety also reporting depressive symptoms [3] and two-thirds of patients with a primary diagnosis of major depressive disorder (MDD) also displaying pathological levels of anxiety [4–6]. It is of importance to distinguish between comorbid anxiety-depression and anxiety or depression occurring alone because the combination has worse health outcomes, entails greater risk of suicide, and is more resistant to treatment [7–9]. Despite being highly debilitating and prevalent, the heritability of this phenotype combination is largely unknown. Similar pharmacotherapies (e.g., SSRI, SNRI) are used to treat anxiety and depression [10, 11], which suggests common biological pathways, but a better description of how genetic factors drive the combination of anxiety and depression is needed to ultimately identify biological causes. Hence, in the current study we aimed to determine whether the genetic contribution to comorbid anxiety and depression differs from depression or anxiety alone.

Meta-analyses report twin-heritability in the range of 30–50% for separate occurrences of anxiety- and depressive disorders [12, 13], indicating a moderate heritable component. Although the shared heritability between these disorder categories is known to be large [14, 15], twin-studies on comorbid anxiety and depression are scarce. In a rare instance, Guffanti et al. [16] estimated the heritability for sequential comorbidity of anxiety disorders and MDD in the range of 53–57% using a relatively small sample of 545 high-risk participants from 65 multigenerational families. The findings by Guffanti et al. suggest that the combination of anxiety and MDD might be more heritable than when each disorder occur alone, but the small sample size together with the probable oversampling of cases warrants replication before firm conclusions can be drawn.

Anxiety- and depressive disorders have also been predicted from polygenic scores (PGS) that are based on genomic variations associated with phenotypes in independent discovery samples [17]. To date, anxiety PGSs explain about 0.5–2.27% of variance when predicting anxiety disorders in independent cohorts [18–21], which is comparable with reports of depression PGSs explaining 0.27–2.2% of variance in depression [22, 23]. Recently, Davies et al. [24] found that those with comorbid anxiety-depression had higher PGSs for Neuroticism and ADHD and lower PGS for educational attainment compared to either condition alone (i.e., no healthy controls were included in the study). The comorbid group also had higher anxiety-PGS compared to depression alone. However, effects were markedly reduced when including additional PGSs in the model, and nullified when including clinical features, trauma exposure, demographics, and additional PGSs in the model. Tentative evidence further suggests that individuals with comorbid anxiety have higher PGSs for a broad depression phenotype (odds ratio per *SD* PGS = 1.17) compared to participants meeting only MDD criteria [25]. These results have been corroborated by recent findings, indicating that depression with comorbid anxiety, as defined by ICD codes, is associated with higher polygenic load for several psychopathological traits in comparison with depression without anxiety [26]. Furthermore, Coombes et al. [27] recently used hospital-based biobanks and diagnostic codes to show that the polygenic contribution, as indexed by PGS for depression, was larger for comorbid anxiety and depression than for each disorder in isolation. Although these reports suggest increased genetic liability for comorbid anxiety and depression compared to when anxiety or depression occur alone, the limited number of studies and the differences in how cases have been ascertained, make generalizable conclusions premature.

Electronic health-record based studies of genetic influences on anxiety and depressive disorders commonly depend on diagnoses from specialist care. This could potentially induce a bias in estimates of genetic liability as most individuals seeking treatment for anxiety or depression initially consult a general practitioner in primary care, and often receive a drug prescription from these practitioners without referral to a specialist. To include patients seeking care for anxiety (*Anxiety-only*), depression (*Depression-only*), or both (*Comorbid* anxiety and depression) from both primary and specialist care, we here used an algorithm to search the Swedish Prescribed Drug Register for information about the symptoms for which drugs were prescribed. This so-called dosage text is written by the GP and contains the main indication for the drug prescription. To validate the ascertainment of cases, we compared which drug classes were most commonly prescribed for each symptom category (Anxiety-only, Depression-only, Comorbid anxiety and depression). In addition to symptom categories based on drug prescription, outcome groups were also defined by International Classification of Diseases (ICD) diagnoses that only included patients referred to specialist care. This allowed us to evaluate whether genetic influences on outcome categories were dependent on the definition of cases (ICD diagnoses vs. drug prescription information). Notably, the term comorbid here refers to both sequential and simultaneous comorbidity where patients have been prescribed drugs for symptoms of anxiety and depression or diagnosed with a depression and an anxiety diagnosis either within a time period or simultaneously [28].

For the purpose of this study, we constructed PGSs for 6 different traits focusing on anxiety, depression, schizophrenia, and neuroticism, while also using 36 predefined PGSs from the polygenic index repository [29]. The first aim was to determine genetic liability for comorbid anxiety and depression, anxiety only, and depression only. We hypothesized that both twin heritability and polygenic contribution would be greater for comorbid anxiety and depression than for anxiety or depression only. A second aim was to determine whether PGSs for anxiety and depression explained more variance in our outcomes than PGSs based on other phenotypes. The third aim was to determine the effect of having extremely high or low PGSs on anxiety, depression or comorbid anxiety and depression to understand if PGSs can be used for patient stratification.

## Method

### Study population

STAGE and YATSS are population cohorts of Swedish twins born between 1959–1985 and 1986–1992, respectively, that are part of the Swedish Twin Registry [30, 31]. The STAGE study invited 25,364 twins to donate saliva for genotyping in 2005–2006, and 6218 twins from YATSS were invited in 2013–2014. Genotyping was performed on the 650 K Illumina Global Screening Array BeadChip for both cohorts in 2017–2019, resulting in a final sample of 13,699 twins with genotype data available for 12,792 individuals. STAGE responders were representative of the Swedish population in terms of educational attainment and family background characteristics [32].

### Ethics

All participants provided written informed consent before submitting their saliva samples to the Swedish Twin Registry, which ensures that only participants with valid consent are included in research. Our study was approved by the Swedish Ethical Review Board (reference number: 2020–00181). All methods were performed in accordance with relevant guidelines and regulations, including the Declaration of Helsinki.

### Measures

#### Anxiety and depression outcomes

We obtained drug prescription data for the entire study population from the Swedish Prescribed Drug Register [33] through the Swedish Twin Registry (STR). We included N06AA non-selective monoamine reuptake inhibitors, N06AB selective serotonin reuptake inhibitors (SSRIs), N06AF monoamine oxidase inhibitors (non-selective), N06AG monoamine oxidase A inhibitors, N06AX other antidepressants (which include certain selective serotonin and noradrenaline reuptake inhibitors as well as other drugs), N05BA benzodiazepine derivatives, N05BE azaspirodecanedione derivatives, R06A antihistamines for systemic use, C07AA beta blocking agents (non-selective), and N03AX other antiepileptics (i.e., pregabalin) based on their Anatomical Therapeutic Chemical code (ATC) [34]. For the above-mentioned ATC-categories, we employed an algorithm that searched through the prescription text, also known as the dosage text, written by the prescribing physician. If the text included any of the words “worry”, “anxiety”, “anxiety issues” (one word in Swedish “orosbesvär”), or “panic”, the drugs were classified as prescribed for symptoms of anxiety. For depression, one of the words “depression”, “depressive”, “dejection” (i.e., “nedstämdhet” in Swedish), or “mood-enhancing” had to be included in the text. The text-searching algorithm resulted in two columns for each drug, one column for symptoms of anxiety and one for symptoms of depression, where individuals were coded as 1 or 0 based on if the drug had been prescribed with or without the relevant target words. Those without prescriptions of the target drugs were coded as NA and included as controls. Thus, individuals seeking non-pharmacological treatment options, such as psychotherapy, are contained within the control group. It is not possible to exclude those that have received non-pharmacological interventions from the control group since medical registries in Sweden are void of information on treatment modality. Available data from internal registries of local authorities show that approximately 1 out 5 seeking primary care for depression- or anxiety disorders received CBT in 2017 [35], but these data include those with multi-treatments (including pharmacological options) and should therefore be interpreted with caution. All drug prescriptions were treated as life-time events and there was no distinction made between individuals prescribed drugs for anxiety and depression simultaneously, meaning the terms occurred in conjunction in the dosage text, or individuals prescribed medication for anxiety on one occasion and depression on another, meaning that the terms occurred in two or more separate dosage texts. Based on the results from the text-searching algorithm, we classified individuals into one of four categories, (1) if the text mentioned both anxiety and depression they were included in the symptom category Comorbid anxiety and depression; (2) if the text mentioned anxiety, but not depression, participants were included in the category Anxiety-only; (3) if the text mentioned depression, but not anxiety, they were included in the category Depression-only; (4) if the text did not mention anxiety or depression they were categorized as *Medication-only*. The last group was included as the target drugs can be prescribed for other indications than anxiety or depression (e.g., otherwise unspecified sleep disorder, premenstrual syndrome, adjustment disorder, PTSD, or chronic pain) or when symptoms of anxiety or depression occur but are not predominant, and physicians sometimes omit stating the indication that the medication is prescribed for. These symptom categories were used as outcomes in subsequent statistical analyses, controls comprised those not included in any of these categories.

We also obtained diagnoses from the National Patient Register, which are assigned by an attending physician based on the International Classification of Diseases, eighth revision (ICD-8) (1969–1986), ninth revision (ICD-9) (1987–1996), or tenth revision (ICD-10) (1997-present). Similar to drug prescriptions, diagnoses were defined as life-time events and treated as binary variables. Participants were categorized in the depression diagnosis group if they had any depression-related ICD code, including 3004 (ICD-8), 300E, 311 (ICD-9), and F32-F39 (ICD-10). Anxiety diagnosis was defined as having at least one of the following codes, 300 (excluding 3003, 3004) (ICD8), 300 (excluding 300D, 300E) (ICD-9), and F40-41 (excluding F41.2), F44-45, F48 (ICD-10). The F41.2 code was excluded from the anxiety diagnosis group, since it refers to having symptoms of both anxiety and depression, but where neither is clearly predominant. Participants with an F41.2 code were grouped in a third category (comorbid depression and anxiety diagnoses), which also included those that had both a depression-related and an anxiety-related ICD code. These 3 groups (Comorbid anxiety and depression, Anxiety-only, Depression-only) were used as separate outcomes in subsequent analyses, in which controls included participants that neither had a diagnosis nor been prescribed any antidepressants or anxiolytic drugs.

#### Polygenic scores (PGS)

A total of 42 PGSs were evaluated. Thirty-six of these came from the polygenic index (PGI) repository, which contains scores derived from large-scale GWASs conducted in 23andMe, UK Biobank [36], as well as other available datasets. For each phenotype, PGSs have been constructed in 11 repository cohorts including the STR, with a leave-one-out approach to distinguish the discovery sample from the target cohort. Hence, PGSs in STAGE and YATSS were created from GWAS summary statistics that exclude these datasets, which allows for unbiased targeting of phenotypes in our sample to test the predictive performance of PGSs from the PGI-repository. Information on quality control and PGS construction have been reported in detail elsewhere [29].

In addition to the 36 PGSs from the PGI-repository, we constructed 6 PGSs based on Lifetime Anxiety Disorder diagnosis (LAD; cases = 31977; controls = 82114), Major Depressive Disorder (MDD; cases = 170756; controls = 329443), schizophrenia (SCZ; cases = 67390; controls = 94015), Neuroticism scores (NEURO; *n* = 323415), the Generalized Anxiety Disorder 7-item scale (GAD-7; *n* = 126175), and the Patient Health Questionnaire-9 (PHQ-9; *n* = 126733). The LAD-PGS was based on summary statistics from the meta-analysis performed by Purves et al. [19], the MDD-PGS was based on summary statistics published in Wray et al. [23] and Howard et al. [37], and the SCZ-PGS was created from summary statistics obtained from Ripke et al. [38]. The NEURO, GAD7, and PHQ9 PGSs were all based on individual level data from the UKB [36, 39]. Ambiguous SNPs (alleles A/T or C/G), trivial SNPs (showed no variation across UKB) and SNPs with a minor allele frequency (MAF) < 0.01 were all excluded. We used the LDAK software [40] with the BLD-LDAK heritability model [41], where contribution of a SNP depends on MAF, linkage disequilibrium, and functional annotations. PGS construction was performed with LDAK-Bolt-Predict for individual-level data as well as LDAK-BayesR-SS for summary statistics and was limited to SNPs that overlapped between cohort genotype, UKB reference panel and GWAS summary statistics. Finally, we converted all PGSs to *z*-scores for interpretation purposes.

### Statistical analyses

#### Twin heritability

Twin heritability (*h*^2^_TWIN_) was estimated by fitting structural equation models that decomposed variance in the outcome measures into additive genetic (*A*), common-environmental (*C*) and unique-environmental variance (*E*) as well as with models that only estimated the A and E components. These models are based on the assumptions that Monozygotic (MZ) and Dizygotic (DZ) twin pairs share common environmental effects to the same extent and that MZ twins share 100% of their segregating alleles, while DZ twins share 50% of their segregating alleles. Thus, the additive genetic effects are shared with correlation equal to 1 between MZ pairs and correlation 0.5 between DZ pairs. Analyses were initially performed using only same sex twin pairs and were then repeated including DZ pairs with different sexes in addition to same sex twin pairs. Due to the case/control design (i.e., binary traits as outcome measures), we used the liability threshold model, which assumes a latent bivariate normal distribution of the observed dichotomous variables [42, 43]. Model fit was assessed by likelihood ratio tests (LRT) and Akaike’s information criteria (AIC). All twin models included sex and age as covariates. Twin heritability was analysed with the mets R-package [44].

#### PGS prediction of anxiety and depression outcomes

To determine effect sizes of PGSs in predicting each of the outcomes based on prescription data (Comorbid anxiety and depression, Anxiety-only, Depression-only, Medication-only) or diagnosis groups (comorbid depression and anxiety diagnoses, anxiety diagnosis, depression diagnosis), we used logistic regression. Effect sizes are presented as adjusted odds ratios (OR) per *SD* PGS, with 95% confidence intervals (95% CI) for each outcome. Pseudo-*R*^2^ (Nagelkerke *R*^2^) was estimated for the full model (all covariates including PGS) and the base model (not including PGS). The latter included sex, age, zygosity (to correct for clustering of family members), the first 20 ancestral principal components (to control for population stratification), and cohort (STAGE, YATSS) as covariates. Proportion of predicted variance was calculated as the difference between the two pseudo-*R*^2^ estimates (Δ*R*^2^). We also calculated area under the Receiver Operating Characteristics curve (AUC) between the base model and the full model including the best performing PGS.

We next included all PGSs in one regression model to assess the ability of each score in predicting the outcomes while controlling for the other PGSs, and evaluated the Δ*R*^2^ to assess how much variance in outcomes could be predicted when all genetic indices were included in the same model. Collinearity diagnostics showed that all variables had a variance inflation factor and tolerance statistic well below 10 and above 0.1, respectively, indicating no significant multicollinearity.

To describe associations across the PGS distribution with each outcome, we first selected the PGSs that had the overall strongest association with the outcomes according to their OR and *p*-value, and divided the sample in deciles based on their PGS. Using logistic regression, we computed the association between each outcome and the highest PGS decile compared with the remaining 80% of the sample (leaving out the bottom 10%). The same analysis was performed for the lowest decile (leaving out the top 10%). These analyses inform on how groups with extreme scores compare with those that have scores closer to the mean. To assess the incremental increase in association between PGS and each outcome across deciles, separate regression analyses were performed for each decile using the lowest as reference.

We performed sensitivity analyses by stratifying the sample based on sex and zygosity, respectively, to assess whether these factors had a significant influence on the results. For all models, *p*-values were corrected for multiple comparisons using the Benjamini-Hochberg procedure [45] for False Discovery Rate (FDR < 0.05).

## Results

### Sample characteristics

All regression models were based on complete cases. Data on drug prescription were retrieved for 13699 individuals, where genotype information was available for 12792 participants (see Table 1 for sample characteristics). Due to missing genotype data, PGS-predictions using regression models with covariates comprised 1028 cases with Comorbid anxiety-depression, 718 cases with Anxiety-only, 484 cases with Depression-only, 1593 cases with Medication-only, with 8969 individuals in the control group. Due to missing data on age and sex (see Table 1), twin heritability analyses were based on 1986 complete MZ-pairs and 1594 DZ-pairs. The control group for analyses of prescription-based outcomes included all individuals without any prescriptions of the target drugs, while analyses of diagnostically defined outcomes included those without drugs or diagnoses in the control group (see supplemental material for the exact *n* in each analysis).

**Table 1:** Sample Characteristics.

Variable	Description	*N*	%, $\bar{{\rm{x}}}$ (*SD*, range)	N06AA-AB	N06AC-AX	Benzo	Antihis	BusProPre	$\bar{{\rm{x}}}$ drugs prescribed
Sample	Total	13699	100%	2425	1140	1064	2133	620	0.54
Sex	Female	7939	58.00%	1830	788	780	1664	458	0.70
	Male	5087	37.10%	595	301	284	469	162	0.36
Age	Age at final data extraction (2023)	13026	51.69 (7.87, 38–85)	–	–	–	–	–	–
Zygosity	Complete MZ	3972	28.99%	695	273	297	655	159	0.52
	Complete DZ same sex	1732	12.64%	305	128	143	262	86	0.53
	Complete DZ diff. Sex	1886	13.77%	253	145	111	223	67	0.42
	No kinship	6109	44.60%	1172	594	513	993	308	0.59
Outcomes	Comorbid Anxiety and Depression	1054	7.07%	978	591	499	784	190	2.89
	Anxiety only	744	4.99%	357	134	307	500	99	1.88
	Depression only	511	3.43%	448	205	44	126	38	1.68
	Medication only	1630	10.93%	642	210	214	723	293	1.28

No kinship = individuals without sibling in data set due to missing genotype information.

*BusProPre* Buspirone or Propranolol or Pregabalin; *Benzo* Benzodiazepines; *Antihis* Antihistamines; *N06AA* non-selective monoamine reuptake inhibitors; *N06AB* selective serotonin reuptake inhibitors; *N06AC-AX* monoamine oxidase inhibitors (non-selective), monoamine oxidase A inhibitors, other antidepressants.

**Fig. 1:**
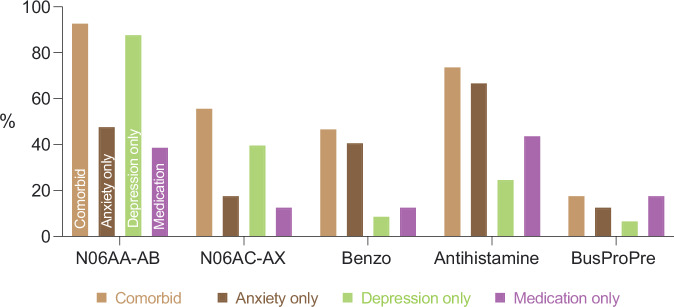
Proportion of outcome groups with prescription divided by drug class. *Note:* N06AA-AB = non-selective monoamine reuptake inhibitors, selective serotonin reuptake inhibitors (SSRIs); N06AC-AX = monoamine oxidase inhibitors (non-selective), monoamine oxidase A inhibitors, other antidepressants (which include certain selective serotonin and noradrenaline reuptake inhibitors as well as other drugs); Benzo = benzodiazepine derivatives; BusProPre = Buspirone, Propranolol, Pregabalin. Drug classifications are based on their Anatomical Therapeutic Chemical code (ATC).

To compare patterns of prescriptions across outcome groups, we computed the proportion of individuals in each group that were prescribed medications from the drug classes N06AA-AB, N06AC-AX, benzodiazepines, antihistamines and Buspirone/Propranolol/Pregabalin (BusProPre) (Fig. 1). In the Anxiety-only category, drugs from the class antihistamines were most frequently prescribed, followed by N06AA-AB and benzodiazepines. In contrast, the Depression-only group had the lowest proportion of prescriptions from the benzodiazepine class compared to any of the other groups. Instead, N06AA-AB was the most commonly prescribed drug class for this group followed by N06AC-AX. The Comorbid anxiety and depression group had high prescription rates of drugs from the classes N06AA-AB and antihistamines as well as benzodiazepines and N06AC-AX. Notably, individuals in the Comorbid symptom category were on average prescribed drugs from more drug classes (2.89) compared to any of the other outcome groups (1.28–1.88). The Medication-only group had the lowest rates of prescriptions (1.28) with antihistamines being the most frequently prescribed, followed by N06AA-AB and BusProPre. These results indicate that the prescription text that was used for categorizing individuals into the outcome groups was associated with different treatment decisions.

To assess diagnoses of anxiety and depressive disorders, we retrieved data from the National Patient Registry. Out of the 1029 participants with an ICD code associated with these disorders, 379 had only a depression diagnosis, 283 had only an anxiety diagnosis, and 367 had comorbid depression and anxiety diagnoses. The group with comorbid anxiety and depression diagnoses consisted of 173 individuals that had both a depression- and an anxiety-related ICD code, 40 individuals with an F41.2 (mixed anxiety and depressive disorder) diagnosis, 50 individuals with F41.2 and an anxiety ICD code, 27 individuals with F41.2 and a depression ICD code, and 77 individuals with F41.2 plus a depression and an anxiety ICD code. As expected, the number of individuals with diagnoses were fewer than the number of individuals with prescriptions, as most individuals receiving prescriptions from primary care are not referred to a specialist that enters a diagnosis in the Swedish Patient Registry.

### Twin-based heritability of anxiety and depression

**Fig. 2:**
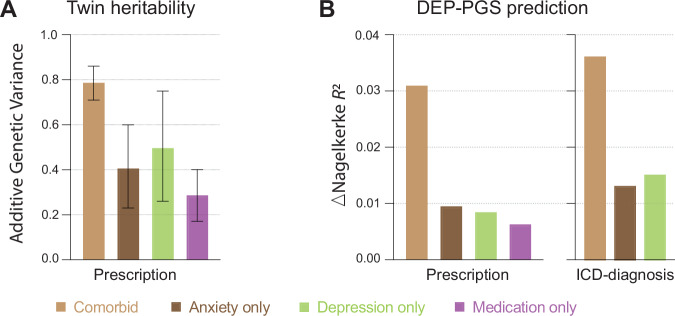
Genetic liability for anxiety and depression outcomes. **A** Twin heritability for each outcome (AE-model), error bars represent 95% CI. **B** Accuracy (Δ*R*^2^) of depressive symptoms PGS (DEP-PGS) from the Polygenic Index Repository in predicting each outcome above the baseline model.

The additive genetic influence on having been prescribed medication for comorbid anxiety and depression was *h*^2^_TWIN_ = 0.79 (95% CI = 0.71–0.86). Lower additive genetic effects were found for having been prescribed medication for Anxiety-only (*h*^2^_TWIN_ = 0.41, 95% CI = 0.23–0.60) or Depression-only (*h*^2^_TWIN_ = 0.50, 95% CI = 0.26–0.75). Further, having been prescribed the same drugs, but without an indication of anxiety or depressive symptoms, resulted in a heritability estimate of *h*^2^_TWIN_ = 0.29 (95% CI = 0.17–0.40). See Fig. 2A and supplementary Table S1 for twin-model estimates.

### Prediction of anxiety and depression from PGSs

#### Regression models with single PGS predictors including covariates

**Fig. 3:**
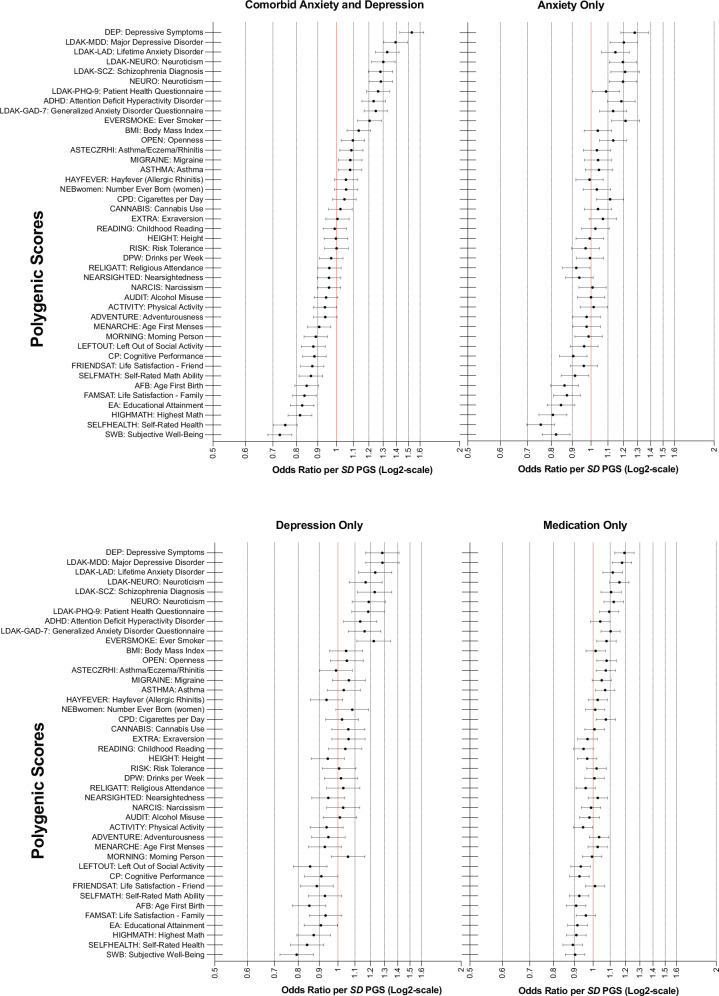
Results from regression models with single PGS predictors including covariates. Adjusted OR per *SD* increase in PGSs predicting prescription-based outcomes. Error bars represent 95% CI.

Figure 3 shows which PGSs that were significantly associated with the different outcomes, and the strength of associations in terms of adjusted OR per *SD* PGS. When modelled as individual predictors, 27 out of the 42 PGSs evaluated (36 from the PGI repository and 6 scores computed for the purpose of this study) showed a significant increase in predicting drugs prescribed for Comorbid anxiety and depression compared with the base model (base model *R*^2^ = 0.041, AUC = 0.632). Out of these 27 PGSs, the PGS for depressive symptoms from the PGI repository (DEP-PGS) was the strongest predictor (Δ*R*^2^ = 0.031, ΔAUC = 0.044, OR = 1.53, 95% CI = 1.43–1.63) followed by the PGS for major depressive disorder (MDD-PGS) computed from Wray et al. [23] using LDAK (Δ*R*^2^ = 0.020, ΔAUC = 0.032, OR = 1.39, 95% CI = 1.30–1.49), PGS for Subjective Wellbeing (SWB-PGS; Δ*R*^2^ = 0.018, ΔAUC = 0.028, OR = 0.73, 95% CI = 0.68–0.78), Lifetime Anxiety Disorder (LAD-PGS) computed with LDAK (Δ*R*^2^ = 0.015, ΔAUC = 0.022, OR = 1.33, 95% CI = 1.25–1.42), PGS for Self-rated Health (Δ*R*^2^ = 0.014, ΔAUC = 0.021, OR = 0.75, 95% CI = 0.70–0.80), and PGS for neuroticism computed with LDAK (Δ*R*^2^ = 0.013, ΔAUC = 0.020, OR = 1.30, 95% CI = 1.22–1.39). The pattern of results was similar for the other outcomes, with DEP-PGS being the best or second-best predictor, but the predictive performance of PGSs was markedly reduced as indicated by lower *R*^2^ values and lower ORs per *SD* PGS (Tables S3, S4 and Fig. 3). The mean value of DEP-PGS (*z*-scores) in the Comorbid anxiety and depression group (mean = 0.334, *SD* = 0.961) was almost double the mean value in the Depression-only (mean = 0.166, *SD* = 0.997) and Anxiety-only (mean = 0.164, *SD* = 0.973) groups. For comparison, mean values were 0.094 (*SD* = 1.01) in Medication-only, and −0.077 (*SD* = 0.993) in the non-medicated control group. One-way ANOVA [*F*(4, 12787) = 54.63, *p* < 2e-16] followed by post-hoc tests, showed that DEP-PGS was on average significantly higher in each outcome group (Comorbid anxiety and depression, Anxiety-only, Depression-only, Medication-only) compared with the non-medicated control group. However, the Comorbid anxiety and depression group also had significantly higher mean DEP-PGS as compared to the other outcome groups, indicating that this is a disorder combination with higher polygenic load than anxiety or depression alone (see Supplemental Table S2 for results of post-hoc tests).

##### Clinical Diagnoses Regressed on Individual PGSs

To validate case classifications based on drug prescriptions, the regression analyses with PGSs as individual predictors were repeated, but this time with the clinical diagnosis groups as outcomes. For the comorbid depression and anxiety diagnoses group (base model *R*^2^ = 0.047, AUC = 0.667), DEP-PGS was the strongest predictor (Δ*R*^2^ = 0.036, ΔAUC = 0.051, OR = 1.70, 95% CI = 1.53–1.90) followed by SWB-PGS (Δ*R*^2^ = 0.030, ΔAUC = 0.048, OR = 0.61, 95% CI = 0.55–0.68) and MDD-PGS (Δ*R*^2^ = 0.026, ΔAUC = 0.038, OR = 1.59, 95% CI = 1.43–1.77). When predicting the probability of having an anxiety diagnosis (base model *R*^2^ = 0.032, AUC = 0.644), LAD-PGS (Δ*R*^2^ = 0.013, ΔAUC = 0.022, OR = 1.41, 95% CI = 1.25–1.60), DEP-PGS (Δ*R*^2^ = 0.013, ΔAUC = 0.016, OR = 1.40, 95% CI = 1.23–1.58) and MDD-PGS (Δ*R*^2^ = 0.012, ΔAUC = 0.018, OR = 1.39, 95% CI = 1.23–1.57) emerged as the top predictors. For the depression diagnosis group (base model *R*^2^ = 0.026, AUC = 0.631), DEP-PGS (Δ*R*^2^ = 0.015, ΔAUC = 0.028, OR = 1.41, 95% CI = 1.27–1.57), MDD-PGS (Δ*R*^2^ = 0.015, ΔAUC = 0.022, OR = 1.40, 95% CI = 1.26–1.55) and SWB-PGS (Δ*R*^2^ = 0.014, ΔAUC = 0.029, OR = 0.71, 95% CI = 0.64–0.79) were the PGSs with the strongest predictive performance (see suppl. Tables S5, S6 for OR, *R*^2^, *N*, and *p*-values for PGSs predicting ICD diagnoses outcomes). DEP-PGS had the overall highest accuracy and predicted more variation in the comorbid anxiety and depression diagnoses group compared with those having only one of these diagnoses, which is consistent with results from the regression analyses with symptom categories defined by drug prescription (see Fig. 2B). Hence, subsequent analyses include only prescription-based symptom categories as outcomes, since they include more cases than the diagnostically defined groups.

#### Regression models with multiple PGS predictors including covariates

In a single regression model containing all 42 PGSs simultaneously along with base-model covariates (base model *R*^2^ = 0.041, AUC = 0.632), only DEP-PGS (OR = 1.23, 95% CI = 1.12–1.35), SCZ-PGS (OR = 1.18, 95% CI = 1.10–1.27), and MDD-PGS (OR = 1.14, 95% CI = 1.06–1.24) remained significantly associated with drug prescription for Comorbid anxiety and depression (Δ*R*^2^ = 0.057, ΔAUC = 0.071). When predicting prescriptions for Anxiety-only (base model *R*^2^ = 0.023, AUC = 0.605), again using all 42 PGSs (Δ*R*^2^ = 0.034, ΔAUC = 0.057), the only significant predictors were SelfHealth-PGS (OR = 0.800, 95% CI = 0.72–0.89) and SCZ-PGS (OR = 1.15, 95% CI = 1.05–1.25). When predicting drug prescription for the Depression-only group (Δ*R*^2^ = 0.031, ΔAUC = 0.046) and the Medication-only group (Δ*R*^2^ = 0.017, ΔAUC = 0.025), no PGS-predictor survived the correction for multiple comparisons (see suppl. Tables S7, S8 for *R*^2^, OR and *p*-values). These results indicate that using multiple PGSs in the same model improves prediction compared to using individual PGSs, but that only a few scores have a unique polygenic contribution. Also, SCZ-PGS seem to have a unique contribution to the genetic influence on the Comorbid anxiety and depression and Anxiety-only categories although not being among the top 5 predictors in the regression models using single PGSs.

#### Sensitivity analyses

To check for possible confounding of zygosity, the sample was split by kinship and all regression analyses were repeated on the subsamples, with no substantial difference in the pattern of results compared to findings based on whole-sample analyses (see suppl. Tables S9–S16 for regression model estimates on split-half samples with no kinship).

Regression models comparing base models with models including PGSs, were repeated on samples stratified for sex. Results show that the best performing PGSs predicted comorbid depression and anxiety (men MDD-PGS: Δ*R*^2^ = 0.018, ΔAUC = 0.044, OR = 1.42, 95% CI = 1.24–1.62; women DEP-PGS: Δ*R*^2^ = 0.042, ΔAUC = 0.074, OR = 1.59, 95% CI = 1.47–1.72) to a greater extent than anxiety only (men SELFHEALTH-PGS: Δ*R*^2^ = 0.006, ΔAUC = 0.000, OR = 0.81, 95% CI = 0.69–0.94; women DEP-PGS: Δ*R*^2^ = 0.020, ΔAUC = 0.056, OR = 1.40, 95% CI = 1.28–1.54) and depression only (men EVERSMOKE-PGS: Δ*R*^2^ = 0.013, ΔAUC = 0.022, OR = 1.38, 95% CI = 1.16–1.66; women DEP-PGS: Δ*R*^2^ = 0.009, ΔAUC = 0.017, OR = 1.28, 95% CI = 1.15–1.43) in both men and women.

#### Outcome associations across the PGS distribution

We next wanted to know how extremely high or low PGSs predict drug prescription, as this could indicate whether scores could be useful to identify groups of vulnerable individuals. The highest and lowest deciles of DEP-PGS, the overall best performing genetic index, were compared with the middle 80% of the sample to estimate ORs. Individuals in the top 10% had greater odds of being prescribed medication for comorbid anxiety and depressive symptoms (OR = 2.02, CI95% = 1.67–2.42), while those in the bottom 10% had a decreased risk (OR = 0.37, CI95% = 0.27–0.51). Hence, individuals with a PGS in one of the extreme ends of the polygenic continuum carry genetic risk or resilience compared to the rest of the distribution (see regression model estimates for all outcomes in suppl. Table S17).

**Fig. 4:**
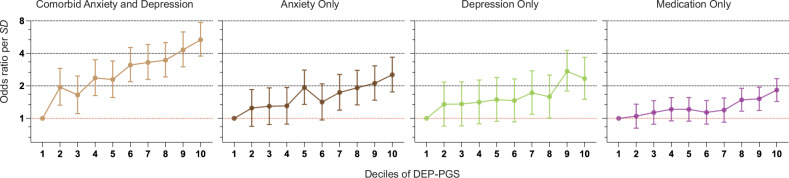
Genetic risk across the PGS distribution. Adjusted OR per SD PGS with 95% CI for each decile of Depressive symptoms PGS (DEP-PGS) compared with the first decile.

To evaluate whether polygenic risk increased linearly across deciles in the distribution, we computed the OR for each decile compared to the lowest decile. We found a cumulative increase in OR with each decile for DEP-PGS (Fig. 4 and suppl. Table S18). These results confirm to expectations from an additive infinitesimal model. The largest OR was found when comparing the lowest and highest deciles of the DEP-PGS (OR = 5.35, 95% CI = 3.78–7.73) in predicting prescription-based symptoms of Comorbid anxiety and depression.

## Discussion

Using polygenic scores and classical twin models, we investigated genetic influences on antidepressant and anxiolytic drugs for comorbid anxiety and depression relative to indications of anxiety or depressive symptoms only. Heritability was 79% for comorbid anxiety and depression, which was larger than for anxiety or depression alone, and is on par with heritability estimates for schizophrenia [46] and bipolar disorder [47]. Comorbid anxiety and depression was also the outcome most strongly associated with PGSs, indicating that SNP-based prediction concurred with twin-based heritability estimates. These results were corroborated by PGSs more accurately predicting comorbid clinical depression and anxiety diagnoses compared with those having only a depression or an anxiety diagnosis. Thus, the results from two complementary methods to evaluate genetic influence, and two different case classification approaches, indicate a stronger genetic influence on the Comorbid symptom category than on anxiety or depressive symptoms alone.

It is well-known that the combination of anxiety and depressive symptoms is more impairing and difficult to treat than each symptom category alone [7–9]. Despite this knowledge, the aetiology of this phenotype combination is poorly understood, and our study represents one of the few attempts made to investigate its genetic basis. There is evidence that the shared genetic component between anxiety and depression is substantially larger than the unique contributions to any of these phenotypes on their own. Large scale GWASs have found robust genetic correlations in the range of 80–95% [18, 19], with 99% of risk variants for anxiety also influencing depression and 73% of risk variants for depression also conferring liability to anxiety [26]. A conclusion from these findings is that the genetic influence on anxiety and depression is best explained by a common genetic factor. Accordingly, the fraction of unique variants that influence each phenotype could, when combined, only represent a small portion of the increase in genetic liability between the Comorbid anxiety and depression outcome compared to Anxiety- or Depression-only outcomes. An important consideration is that the large genetic overlap between anxiety and depression has been estimated in GWASs employing minimal phenotyping approaches that do not consider phenotypic heterogeneity [22]. Recent findings based on more refined phenotyping showed that MDD with comorbid anxiety was twice as heritable compared to a broadly defined depression phenotype [48], indicating that there may be unique subsets of genetic variants associated with certain disorder subtypes not captured by previous GWASs. Thus, performing a large scale GWASs of the Comorbid anxiety and depression symptom category could be of value, as it would probably increase the predictive power of PGS predictions and help to tease out whether loci associated with this phenotype combination differs from MDD or anxiety disorders alone.

In line with the reported large genetic correlations between anxiety and depression [18, 19, 26], PGS for depressive symptoms predicted Anxiety-only to the same extent as Depression-only. The same pattern emerged for the PGS for lifetime anxiety diagnosis (LAD-PGS) that did not predict anxiety better than depression. PGSs for psychiatric disorders (anxiety, depression, schizophrenia, and ADHD) were however better at predicting anxiety, depression and their combination than were PGSs for physical disorders, body length or consumption of alcohol or marijuana. PGSs for subjective wellbeing and cognitive measures in turn predicted resilience.

When controlling for all PGSs, the PGS for schizophrenia emerged as a significant predictor for the Anxiety-only and Comorbid symptom categories, suggesting that this PGS includes unique risk variants common to several disorders. When including all PGSs (and covariates) in one model, we observed a reduction in effect size estimates for each PGS compared to models with single PGS predictors and covariates, which is similar to what Davies et al. [24] reported in their study. The decrease in effect size estimates for one PGS when controlling for the others is due to overlap in genetic variants and thus correlations between different PGSs in the model. The potential effects of other relevant covariates such as clinical features and trauma exposure to the predictive performance of PGSs predicting case/control status for Comorbid anxiety need to be tested in future studies. However, in line with results from models using single PGS predictors and covariates, the Δ*R*^2^ for the model with all PGSs (and covariates) is almost twice as large for the comorbid group compared with depression or anxiety alone. Hence, regardless of whether models include one or several PGSs, the genetic liability is greater for Comorbid anxiety and depression compared to each disorder alone.

The finding of a distinction between the genetic contribution to comorbid anxiety and depressive symptoms relative to their isolated manifestations could reflect a more general genetic liability for psychopathology. In a meta-analysis across eight disorders, the Cross Disorder Group of the Psychiatric Genomics Consortium found 109 pleiotropic loci associated with two or more disorders [49], which extends previous results of shared common variant risk across schizophrenia, MDD, bipolar disorder, anxiety disorders, and ADHD [50]. Thus, the high degree of genetic overlap among psychiatric disorders suggests that clinical classifications of discrete disorder categories do not reflect distinct underlying processes at the genetic level. A dimensional account of psychopathology has suggested that most common psychiatric disorders are unified by a single transdiagnostic dimension representing lesser-to-greater severity of psychopathology, referred to as the p-factor [51]. Comorbid anxiety and depression represent greater severity than anxiety or depression alone, and the stronger genetic influence on the comorbid anxiety and depression outcome could be a result of a genetic factor that makes individuals prone to developing psychiatric illness in general [52]. Extending this line of thought to other psychiatric diagnoses, it could be predicted that the combination of any two diagnostic or symptom categories would be associated with greater heritability than each diagnosis in isolation. This hypothesis could be tested in future research.

Increasing the predictive performance of PGSs is important for risk stratification. We here found that the odds of having symptoms of anxiety and depression was more than 5 times larger in the top decile of DEP-PGS compared to the lowest. This finding does not directly convey clinical utility but could be useful in combination with clinical features. Adding other omics data than PGSs, such as metabolomics or proteomics data, could increase the predictive accuracy to a level at which it could be used to identify groups of people where preventive interventions would be motivated. We also employed AUC estimates to index how well PGSs discriminated between participants with or without depression and anxiety. Participants with comorbid depression and anxiety determined from prescription data were distinguished from participants without depression or anxiety with an AUC of 68% (i.e., 18% above chance level of 50%) based on DEP-PGS, sex, age and ancestral principal components. The DEP-PGS by itself contributed 4.4% to the overall classification accuracy of the model. When comorbid depression and anxiety was defined by ICD diagnoses instead of from prescription data, the AUC was 72% (5.1% unique contribution by DEP-PGS). An AUC of 70% is generally considered fair [53] and a 4–5% increase in overall accuracy solely based on one polygenic score is an important improvement in predictive performance, but questionable for the purpose of risk screening in the general population. To determine a cut-off in terms of AUC for when predictive factors should be implemented in screening and preventive treatment efforts is complex and depends on factors such as patient benefits, costs for the health care system and eventual risks associated with the intervention. For comparison, population screening for adverse childhood events, a well-known social risk factor for psychiatric disorders [54–56] is already underway in the USA where individuals with high scores on an adverse childhood events inventory are referred for health interventions [57]. Adverse childhood event scores have been shown to predict anxiety with an AUC ranging from 54% to 59%, and depression with an AUC between 57% and 62% depending on whether adverse childhood events were assessed prospectively or retrospectively [58], which underscores that even modest AUCs can justify interventions if the patient benefits are judged to be large enough. Thus, if PGSs predicting anxiety and depression in the future also can be shown to predict responses to preventive interventions, they could be useful in guiding treatment decisions to improve patient health. We further foresee that PGSs are likely to complement other features in panels of prognostic markers as their predictive performance will increase.

The study had limitations. Depression related PGSs were based on larger discovery samples than anxiety-related scores, which impacts the results, as the out-of-sample predictive performance of a PGS is dependent on the definition of the phenotype, number of cases, and size of the discovery sample [25]. Therefore, predictive performance will likely increase with larger samples combined with more refined phenotyping. Case classifications in GWASs have generally not considered comorbidity and the summary statistics underlying the construction of anxiety and depression related PGSs used in our study likely included a fair proportion of comorbid cases, which might have contributed to better predictive accuracy for comorbid anxiety and depression compared with anxiety or depression alone. However, this does not explain why the twin-based estimates showed the same pattern of results with a larger heritable component compared with the other outcome groups, and the concordance of findings speaks to the validity of our conclusions despite the current limitations. Outcomes were based on symptom categories and treated as life-time events. Consequently, we do not know if individuals’ anxiety and depression co-occurred or whether they were separated in time in the group with comorbid anxiety and depressive symptoms. In either case, our results point to the genetic propensity of having been prescribed drugs for comorbid anxiety and depressive symptoms or having been diagnosed with both disorders in a clinical setting.

In conclusion, we report larger genetic influence on comorbid anxiety and depressive symptoms than on either symptom category alone. A tentative explanation could be that depression and anxiety can be described by one latent variable and that increasing severity along this latent variable is associated with increased genetic liability.

## Supplementary information

Supplemental Tables

## Data Availability

The STR is responsible for the data and any access, private or public, needs to be preceded by a formal application to STR. Data is currrently being stored at Uppsala Multidisciplinary Center for Advanced Computational Science (UPPMAX). R code is available at request.
